# Genome-wide identification and analysis of *Catharanthus roseus* RLK1-like kinases in *Nicotiana benthamiana*

**DOI:** 10.1186/s12870-021-03208-x

**Published:** 2021-09-18

**Authors:** Shaofei Rao, Xinyang Wu, Hongying Zheng, Yuwen Lu, Jiejun Peng, Guanwei Wu, Jianping Chen, Fei Yan

**Affiliations:** 1 State Key Laboratory for Managing Biotic and Chemical Threats to the Quality and Safety of Agro-products, Key Laboratory of Biotechnology in Plant Protection of Ministry of Agriculture and Zhejiang Province, Institute of Plant Virology, Ningbo University, Ningbo, 315211 China; 2 College of Life Science, China Jiliang University, Hangzhou, 310058 China

**Keywords:** *Catharanthus roseus* RLK1-like kinase, *Nicotiana benthamiana*, Genome-wide, Expression analysis

## Abstract

**Background:**

The *Catharanthus roseus* RLK1-like kinase (CrRLK1L) is a subfamily of the RLK gene family, and members are sensors of cell wall integrity and regulators of cell polarity growth. Recent studies have also shown that members of this subfamily are involved in plant immunity. *Nicotiana benthamiana* is a model plant widely used in the study of plant-pathogen interactions. However, the members of the NbCrRLK1L subfamily and their response to pathogens have not been reported.

**Results:**

In this study, a total of 31 CrRLK1L members were identified in the *N. benthamiana* genome, and these can be divided into 6 phylogenetic groups (I-VI). The members in each group have similar exon-intron structures and conserved motifs. NbCrRLK1Ls were predicted to be regulated by cis-acting elements such as STRE, TCA, ABRE, etc., and to be the target of transcription factors such as Dof and MYB. The expression profiles of the 16 selected *NbCrRLK1Ls* were determined by quantitative PCR. Most *NbCrRLK1Ls* were highly expressed in leaves but there were different and diverse expression patterns in other tissues. Inoculation with the bacterium *Pseudomonas syringae* or with *Turnip mosaic virus* significantly altered the transcript levels of the tested genes, suggesting that *NbCrRLK1Ls* may be involved in the response to pathogens.

**Conclusions:**

This study systematically identified the CrRLK1L members in *N. benthamiana*, and analyzed their tissue-specific expression and gene expression profiles in response to different pathogens and two pathogens associated molecular patterns (PAMPs). This research lays the foundation for exploring the function of NbCrRLK1Ls in plant-microbe interactions.

**Supplementary Information:**

The online version contains supplementary material available at 10.1186/s12870-021-03208-x.

## Background

Receptor-like kinases belong to the RLK/Pelle gene family, which contains more than 600 members in the Arabidopsis genome and can be divided into 46 subfamilies. It plays an important role in plant growth, development and defense responses [1, 2]. RLKs are composed of an extracellular domain (ECD), a transmembrane domain and an intracellular kinase domain [3]. The ECDs of plant RLKs are very diverse, and may include extensin-like domains, lectin-like domains, epidermal growth factor-like domains, lysine motifs, and leucine-rich repeat (LRR) domains [4]. RLK ECDs can undergo homologous or heterologous oligomerization to sense endogenous or exogenous ligands, including peptides, steroids, oligosaccharides, polysaccharides, and lipopolysaccharides, and transduce these signals to the inside of cells [3, 5, 6].

Among the different clade members of plant RLKs, the *Catharanthus roseus* receptor-like kinase 1-like proteins (CrRLK1Ls) have received extensive attention in the past two decades because they regulate cell wall integrity [7]. Members of this subfamily are very conserved among all plants currently analyzed, including mosses and liverworts, indicating that the subfamily has an ancient origin [8]. CrRLK1L contains a carbohydrate binding domain (called malectin-like domain because it is similar to the malectin protein in animals), a transmembrane helix, and a C-terminal intracellular serine threonine kinase domain. The Arabidopsis genome contains 17 members, most of which have been functionally identified [7, 9]. The widely studied member of CrRLK1L is the ubiquitously expressed FERONIA (FER). FER is necessary to recognize the pollen tube in female gametes. In synergids, FER is located at the filiform apparatus and is required to maintain the communication between the synergids and the pollen tube [10]. In addition, FER has growth-related functions in different types of cells in plants. For example, FER can regulate the apical growth of cells, including trichomes and root hairs [11]. FER also regulates the morphology of leaf epidermal cells by affecting the formation of lobes [12]. ANXUR1 (ANX1) and ANXUR2 (ANX2), which are pollen-specific and functionally redundant, are located on the plasma membrane at the tips of the pollen tube. Although they have the highest homology with FER, their function and tissue specificity are completely different [13, 14]. ANX1 and ANX2 are necessary to maintain the integrity of the pollen tube during the apical polar growth process. The pollen tubes of an *anx1 anx2* double mutant ruptured prematurely, leading to male sterility [13, 14]. A recent study showed that the other two redundant members of the CrRLK1L subfamily expressed in pollen, BUDDHA’S PAPER SEAL1 (BUPS1) and BUPS2, are also necessary for the integrity of the pollen tube. BUPS1 and BUPS2 form complexes with ANX1 and ANX2, and both of them can bind to RALF4 and RALF19 expressed by pollen [15]. THESEUS1 (THE1) is reported to monitor the cell wall status. In the damaged cell wall environment, plants activate a THE1-dependent pathway to inhibit growth [16]. ERU is a positive regulator of cell growth and is very important for cytoplasmic NH4^+^ balance [17]. CURVY1 (CVY1), another CrRLK1L, plays an important role in the morphogenesis of trichome and tapetal cells, the transition from trophic state to reproductive state, and seed production [18]. CAP regulates the growth of calcium-dependent pollen tubes and participates in maintaining the composition of root hair cell walls during root tip growth [16, 19]. The other four CrRLK1Ls, MEDOS1–4 are related to growth regulation in response to the presence of metal ions [20].

RAPID ALKALINIZATION FACTOR (RALF) peptides are reported to be ligands for some members of CrRLK1L [21, 22]. These peptides are widely distributed in terrestrial plants, and their activities are related to pH modulation and the production of reactive oxygen species (ROS) [22–24]. The RALF family has about 34 members in Arabidopsis, and they are differentially expressed in different plant tissues [25]. RALF1 treatment can inhibit the root and hypocotyl elongation of Arabidopsis seedlings [26, 27]. Recent studies have shown that RALF1 can directly bind to the extracellular domain of FER and induce the phosphorylation of proton pump AHA2, which is essential for the extracellular alkalinization and cell growth regulation induced by RALF1 [22]. RALF17, RALF23, RALF33, and RALF32 have also been shown to regulate FER. Similar to RALF1, RALF23, RALF33, and RALF32 can induce FER-dependent seedling growth inhibition, and both RALF23 and RALF33 negatively regulate plant immunity [21]. Interestingly, RALF23 can inhibit the formation of FLAGELLIN-SENSING2 (FLS2)/ BRI1-ASSOCIATED RECEPTOR KINASE1 (BAK1) and ELONGATION FACTOR TU RECEPTOR (EFR)/ BAK1 complexes induced by pathogen-associated molecular patterns (PAMPs) flg22 and elf18 respectively through FER [21]. Recent structural and biochemical data indicate that LORELEI (LRE)-LIKE GLYCOSYLPHOSPHATIDYLINOSITOL (GPI)-ANCHORED PROTEIN 1 (LLG1) or LLG2 can directly bind to RALF23 to form a RALF23-LLG1/2-FER heteropolymer complex, and other RALFs sharing a conserved N-terminal region with RALF23 may be perceived in a similar manner [28]. Ge et al. showed that RALF4 and RALF19 can bind to the extracellular domain of ANX1, ANX2, BUPS1 and BUPS2 with high affinity, indicating that these peptides are ligands of the ANX1/2-BUPS1/2 receptor complex [15].

Recent studies have shown that members of the CrRLK1L subfamily also have some roles in plant immunity. *fer-2* and *fer-4* mutants are less sensitive to ROS accumulation induced by efl18 and flg22, and are more susceptible to *Pseudomonas syringae* pv. *tomato* DC3000 (*Pst* DC3000 COR^−^) infection. FER helps ligand-induced immune receptor FLS2, EFR and their co-receptor BAK1 to form a complex to initiate immune signals and positively regulate PAMP-trigered immunity [21]. It has been reported that FER can also inhibit JA and COR signals by phosphorylation and destabilization of MYC2, the main regulator of JA signalling, and positively regulates plant immunity. RALF23 stabilizes MYC2 and enhances JA signalling through FER to negatively regulate immunity [29]. ANX1 and ANX2 in Arabidopsis have been shown to negatively regulate the immune response mediated by Pattern Recognition Receptor (PRR) and Nod-Like Receptor (NLR). ANX1 binds constitutively with the bacterial flagellin receptor FLS2 and its co-receptor BAK1. After FLS2 senses flagellin, it can promote the binding of ANX1 and BAK1, so it will interfere with the formation of the FLS2-BAK1 complex to weaken the PRR signal [30]. In addition, ANX1 can form a complex with NLR protein RESISTANT TO PSEUDOMONAS SYRINGAE2 (RPS2) and RESISTANCE TO *P. SYRINGAE* PV MACULICOLA1 (RPM1), and ANX1 can promote the degradation of RPS2 to reduce RPS2-mediated cell death [30]. Another CrRLK1L member, LET1, was recently discovered to regulate the autoimmune pathway of *mekk1-mkk1/2-mpk4* by forming a complex with the suppressor of *mkk1 mkk2* 2 (SUMM2) and MAP kinase kinase kinase 2 (MEKK2). The complex formed by MEKK2, LET1, and SUMM2 can fight against the ubiquitination and degradation of SUMM2 mediated by the F-box protein CPR1, thereby regulating the accumulation and activation of SUMM2 [31].

At present, members of CrRLK1L in Arabidopsis, *Oryza sativa*, *Gossypium*, *Populus trichocarpa*, *Fragaria vesca* and other plants have been identified [1, 32–36]. *Nicotiana benthamiana* is widely used to study plant-pathogen interactions. However, the members of CrRLK1L in *N. benthamiana* have not been identified, and their response to different pathogens has not been reported. This study identified the members of CrRLK1L in *N. benthamiana*, and analyzed their tissue-specific expression patterns and gene expression profiles in response to different pathogens and two PAMPs. This research lays the foundation for further research on the function of NbCrRLK1L in plant-microbe interactions.

## Results

### Genome-wide identification and naming of NbCrRLK1L members

CrRLK1L contains an extracellular malectin-like domain, a transmembrane helix, and an intracellular kinase domain. We identified possible CrRLK1L members in *Nicotiana benthamiana* based on these criteria. The amino acid sequences of the 17 identified Arabidopsis CrRLK1L members were downloaded from TAIR (http://www.arabidopsis.org/), and the *N. benthamiana* genome sequence was downloaded from Sol Genomics Network (https://solgenomics.net/). After two rounds of BLASTP, 31 NbCrRLK1Ls were identified (Additional file 1: Table S1). These members were named NbCrRLK1L1 to NbCrRLK1L31 according to their chromosome locations (Table 1). The vast majority of members contain about 800 amino acids. The largest contains 954 aa, and the smallest only 439 aa. Their molecular weights are between 50 and 106 kDa, and their theoretical isoelectric points range between 5.3 and 6.54 (Table 1).

**Table 1:** Detailed information of the 31 predicted CrRLK1Ls proteins in *N. benthamiana*

Gene Symbol	Gene Locus	Gene Position	Chromosome Location	Strand	CDS (bp)	Protein Length (aa)	Molecular weight (kDa)	Theoretical PI
*NbCrRLK1L1*	Niben101Scf00063g05010.1	Niben101Scf00063	Niben101Scf00063:516491,519040	–	2547	849	94.20	5.99
*NbCrRLK1L2*	Niben101Scf00687g08007.1	Niben101Scf00687	Niben101Scf00687:834396,837032	–	2634	878	97.23	5.78
*NbCrRLK1L3*	Niben101Scf01018g02004.1	Niben101Scf01018	Niben101Scf01018:258892,264285	+	2712	904	100.87	5.71
*NbCrRLK1L4*	Niben101Scf01025g13013.1	Niben101Scf01025	Niben101Scf01025:1432073,1434643	–	2568	856	94.68	5.27
*NbCrRLK1L5*	Niben101Scf01061g08039.1	Niben101Scf01061	Niben101Scf01061:950012,952540	–	2526	842	93.45	5.89
*NbCrRLK1L6*	Niben101Scf01075g00017.1	Niben101Scf01075	Niben101Scf01075:37046,47016	–	1317	439	50.15	5.75
*NbCrRLK1L7*	Niben101Scf01395g00019.1	Niben101Scf01395	Niben101Scf01395:28554,42854	+	2862	954	106.06	5.30
*NbCrRLK1L8*	Niben101Scf01934g04017.1	Niben101Scf01934	Niben101Scf01934:510898,513583	–	2388	796	87.87	6.30
*NbCrRLK1L9*	Niben101Scf01976g00011.1	Niben101Scf01976	Niben101Scf01976:181105,183618	–	2511	837	91.68	5.42
*NbCrRLK1L10*	Niben101Scf01992g01013.1	Niben101Scf01992	Niben101Scf01992:157667,160823	–	2652	884	96.92	5.33
*NbCrRLK1L11*	Niben101Scf02441g04008.1	Niben101Scf02441	Niben101Scf02441:525793,528408	–	2613	871	96.68	6.03
*NbCrRLK1L12*	Niben101Scf02449g02004.1	Niben101Scf02449	Niben101Scf02449:261360,277268	–	2124	708	78.65	5.88
*NbCrRLK1L13*	Niben101Scf02639g04006.1	Niben101Scf02639	Niben101Scf02639:387053,389425	+	2370	790	88.96	5.58
*NbCrRLK1L14*	Niben101Scf02712g00006.1	Niben101Scf02712	Niben101Scf02712:14349,16844	–	2493	831	91.49	5.37
*NbCrRLK1L15*	Niben101Scf02793g03001.1	Niben101Scf02793	Niben101Scf02793:351588,355656	–	2190	730	81.73	6.86
*NbCrRLK1L16*	Niben101Scf03407g02009.1	Niben101Scf03407	Niben101Scf03407:214538,217054	–	2514	838	92.55	5.77
*NbCrRLK1L17*	Niben101Scf03747g00006.1	Niben101Scf03747	Niben101Scf03747:58947,61529	–	2580	860	96.20	5.53
*NbCrRLK1L18*	Niben101Scf03794g01015.1	Niben101Scf03794	Niben101Scf03794:244820,248030	+	2301	767	85.21	6.51
*NbCrRLK1L19*	Niben101Scf03905g01001.1	Niben101Scf03905	Niben101Scf03905:172169,174751	+	2580	860	95.29	6.69
*NbCrRLK1L20*	Niben101Scf03939g00003.1	Niben101Scf03939	Niben101Scf03939:45922,48414	+	2490	830	91.39	5.94
*NbCrRLK1L21*	Niben101Scf04123g01031.1	Niben101Scf04123	Niben101Scf04123:265055,267721	–	2664	888	96.81	6.09
*NbCrRLK1L22*	Niben101Scf04861g00003.1	Niben101Scf04861	Niben101Scf04861:29496,32135	+	2637	879	96.93	5.77
*NbCrRLK1L23*	Niben101Scf05300g01006.1	Niben101Scf05300	Niben101Scf05300:186398,188962	–	2562	854	94.45	5.40
*NbCrRLK1L24*	Niben101Scf05928g00006.1	Niben101Scf05928	Niben101Scf05928:42110,44983	+	2559	853	94.72	5.67
*NbCrRLK1L25*	Niben101Scf07442g01027.1	Niben101Scf07442	Niben101Scf07442:255193,257823	–	2628	876	97.05	6.15
*NbCrRLK1L26*	Niben101Scf07619g00006.1	Niben101Scf07619	Niben101Scf07619:26955,29621	–	2664	888	96.62	6.02
*NbCrRLK1L27*	Niben101Scf08500g01001.1	Niben101Scf08500	Niben101Scf08500:153954,156035	+	2079	693	77.39	8.91
*NbCrRLK1L28*	Niben101Scf10015g05008.1	Niben101Scf10015	Niben101Scf10015:550054,552437	–	1947	649	72.03	6.33
*NbCrRLK1L29*	Niben101Scf10015g10004.1	Niben101Scf10015	Niben101Scf10015:1109772,1112147	+	2373	791	88.78	5.71
*NbCrRLK1L30*	Niben101Scf25547g00009.1	Niben101Scf25547	Niben101Scf25547:10845,21227	–	2697	899	100.02	5.30
*NbCrRLK1L31*	Niben101Scf25768g00010.1	Niben101Scf25768	Niben101Scf25768:9117,12488	–	2640	880	100.02	6.54

### Phylogenetic analysis

**Fig. 1:**
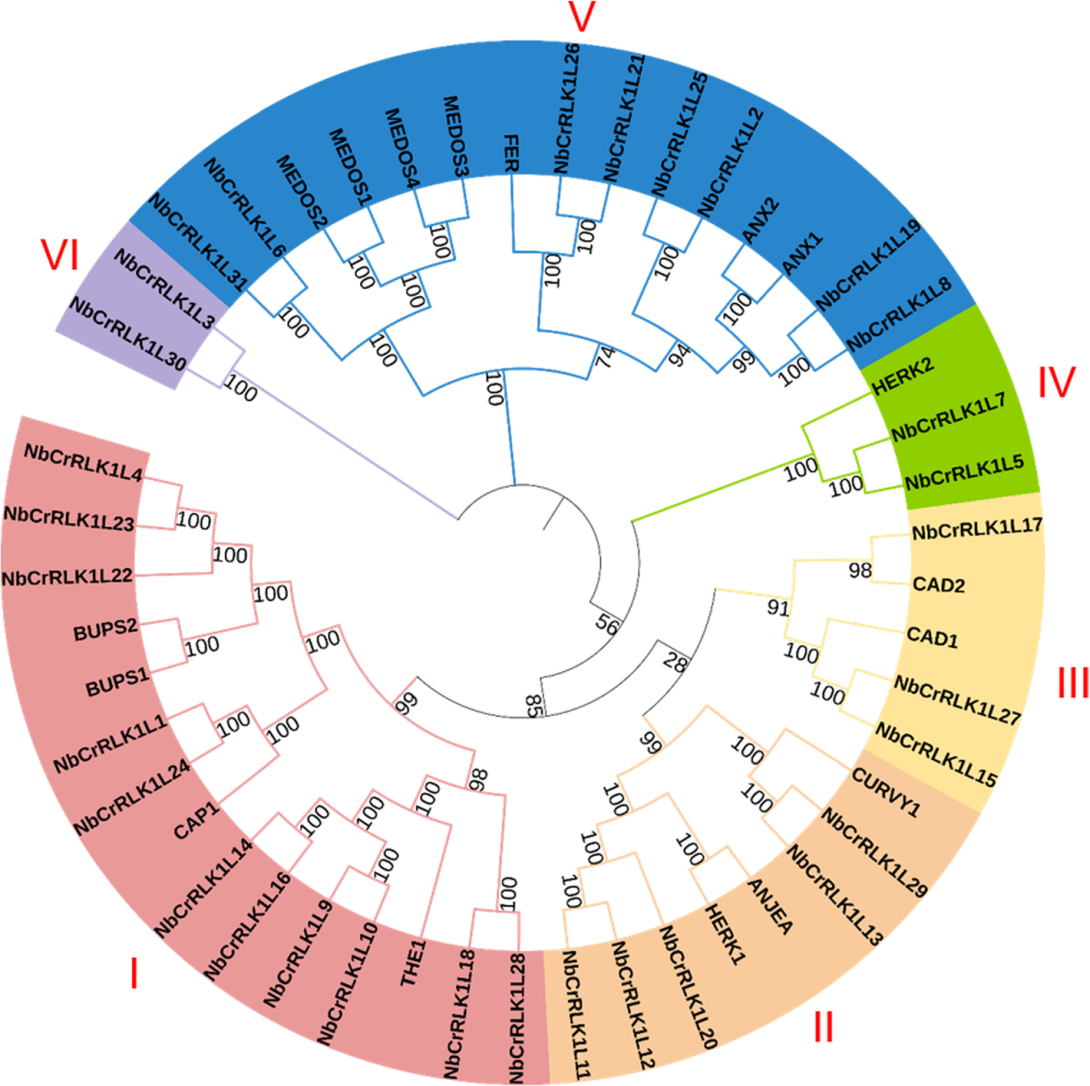
Phylogenetic tree of malectin-like domain-containing proteins in *N. benthamiana* and *A. thaliana.* The phylogenetic tree was constructed using CrRLK1L amino acid sequences by the neighbor-joining method in MEGA X with 1000 bootstrap replicates. The phylogenetic tree was divided into six groups, which are shown in different colors, and identified by red Roman numerals

In order to better understand the evolutionary relationship of the CrRLK1L subfamily genes in Arabidopsis and *N. benthamiana*, the amino acid sequences of 17 Arabidopsis and 31 *N. benthamiana* members were used to construct a phylogenetic tree. Based on the classification of CrRLK1Ls in rice [32], the 31 NbCrRLK1L genes could be divided into 6 groups. As shown in Fig. 1, there are 11 members in group I (NbCrRLK1L4, − 23, − 22, − 1, − 24, − 14, − 16, − 9, − 10, − 18, − 28), 5 members in group II (NbCrRLK1L11, − 12, − 20, − 13, − 29), 3 members in group III (NbCrRLK1L15, − 27, − 17), 2 members in group IV (NbCrRLK1L5, − 7), 8 members in group V (NbCrRLK1L8, − 19, − 2, − 25, − 21, − 26, − 6, − 31), and 2 members in group VI (NbCrRLK1L3, − 30). At least one CrRLK1L from *Arabidopsis thaliana* was included in each group except VI. The first and fifth groups have the most members, indicating that members in different groups have experienced different evolutionary events.

### Analysis of NbCrRLK1L conserved motifs, gene structure and domains

**Fig. 2:**
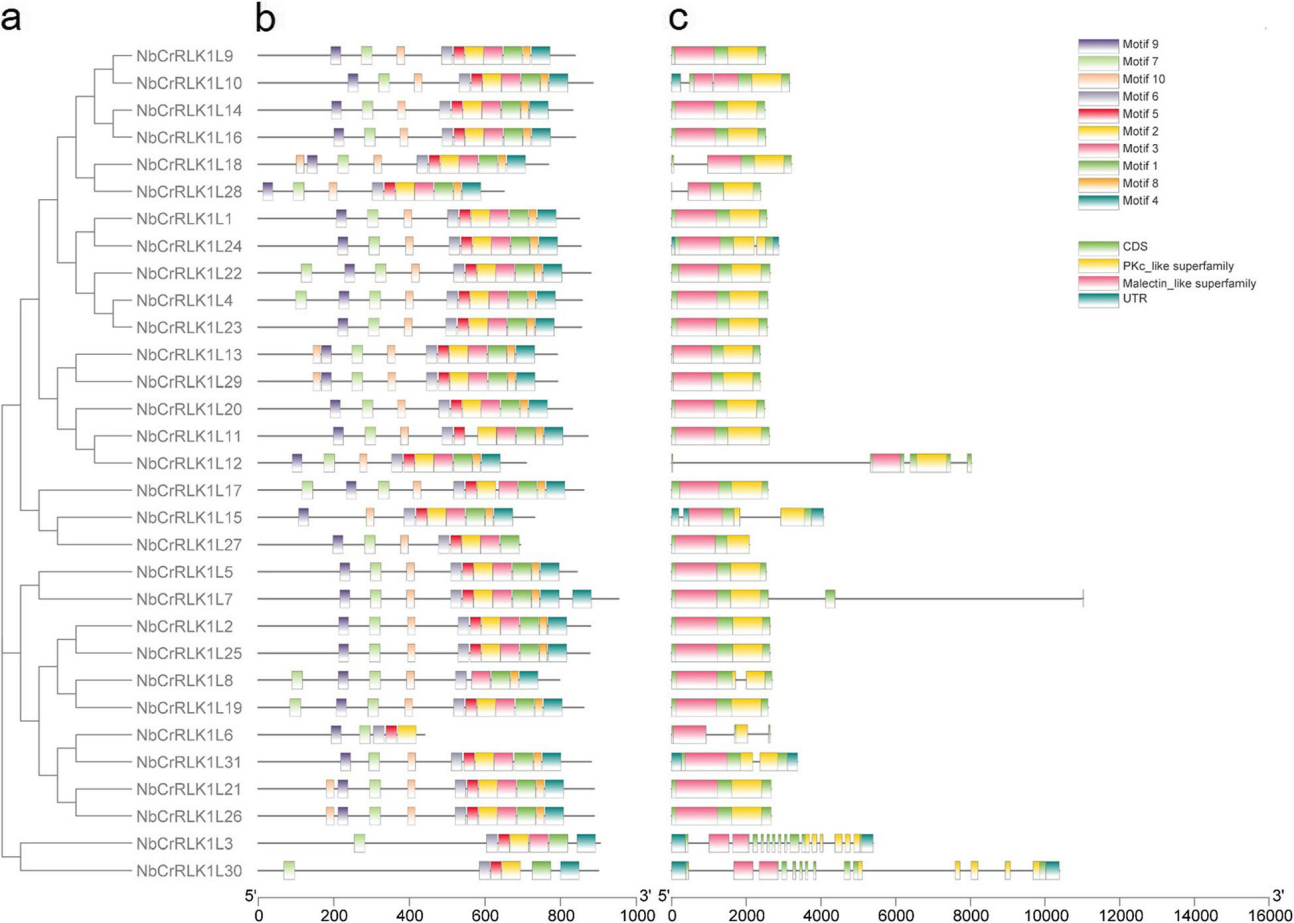
Analysis of conserved motifs, gene structure and domains in the *CrRLK1L* genes of *N. benthamiana.* **a** Phylogenetic tree constructed using the NbCrRLK1L protein sequences. **b** 10 types of conserved motifs predicted in the NbCrRLK1L protein sequences. The different motifs are shown in different color boxes. The sequence information for each motif is provided in Additional file 2: Table S2. **c** The gene structure of NbCrRLK1L members. Untranslated regions, exons, and introns are shown as light blue boxes, light green boxes and horizontal lines, respectively. The red and yellow boxes represent the Malectin-Like Domain (MLD) and Protein Kinase Domain (PK) respectively

To further understand the evolution of CrRLK1L members, we compared conserved motifs, functional domains and the exon-intron organization of NbCrRLK1L members. An online MEME analysis was used to identify motifs among the 31 NbCrRLK1L members, and a total of 10 conserved motifs were predicted (Additional file 2: Table S2). The distribution of NbCrRLK1L motifs is relatively similar (Fig. 2a and b). Most members contain 9–10 motifs, except for NbCrRLK1L6, which has only five motifs. NbCrRLK1L27 contains 8 motifs; NbCrRLK1L3 and NbCrRLK1L30 contain 7 and 6 motifs respectively (Fig. 2b). Most members of *NbCrRLK1L* have no introns (Fig. 2c), consistent with the findings from other plants including Arabidopsis, rice, poplar, strawberry, cotton and legumes [24–28]. Five members (*NbCrRLK1L 18*, − *28*, − *24*, − *8*, − *31*) have a single intron, while *NbCrRLK1L3* and *NbCrRLK1L30*, have multiple introns. All 31 members have the conserved malectin-like domain and the protein kinase domain (Fig. 2c).

### Prediction of promoter cis elements and transcription factors

**Fig. 3:**
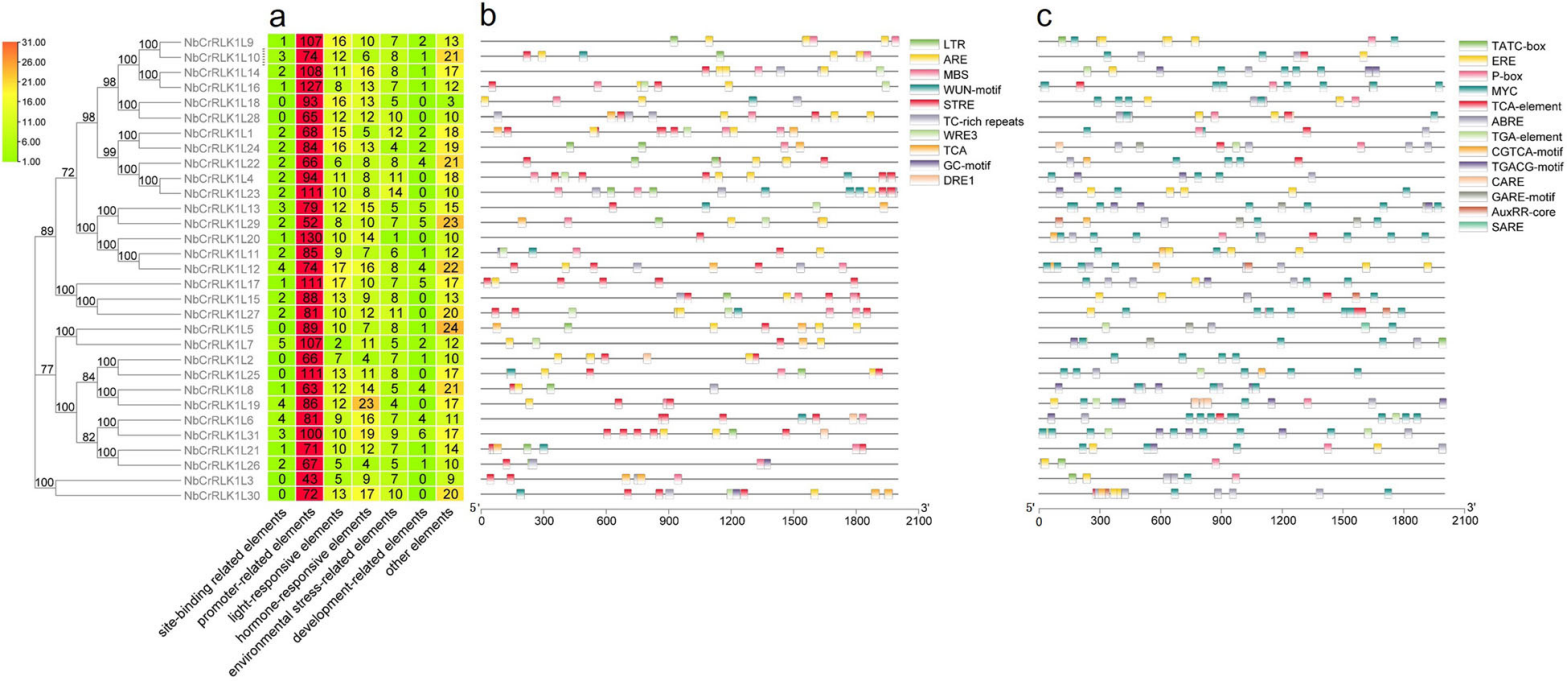
Prediction of cis-acting elements in the *NbCrRLK1L* promoter regions. **a** Schematic representation of the numbers of seven types of cis-acting elements predicted in the promoter region of each *NbCrRLK1L* member. **b** - **c** The type, quantity and position of environmental stress-related elements (**b**) and hormone-response elements (**c**) in the *NbCrRLK1L* promoter region

The cis-acting elements of the *NbCrRLK1L* promoter region were analyzed, and a total of 4154 cis-acting elements of 83 types were predicted (Additional file 3: Table S3). These cis-acting elements are related to environmental stress, hormonal response, development, light response, site binding, promoters, and other functions. The most numerous elements are promoter-related elements, with 43–130 in each *NbCrRLK1L* (Fig. 3a). A total of 229 elements related to environmental stress were predicted in 10 categories, the largest numbers of which were STRE, TCA and ARE elements (Fig. 3b). A total of 352 hormone-related components were predicted in 13 categories, mainly related to ABA, JA, GA, and auxin (Fig. 3c).

**Fig. 4:**
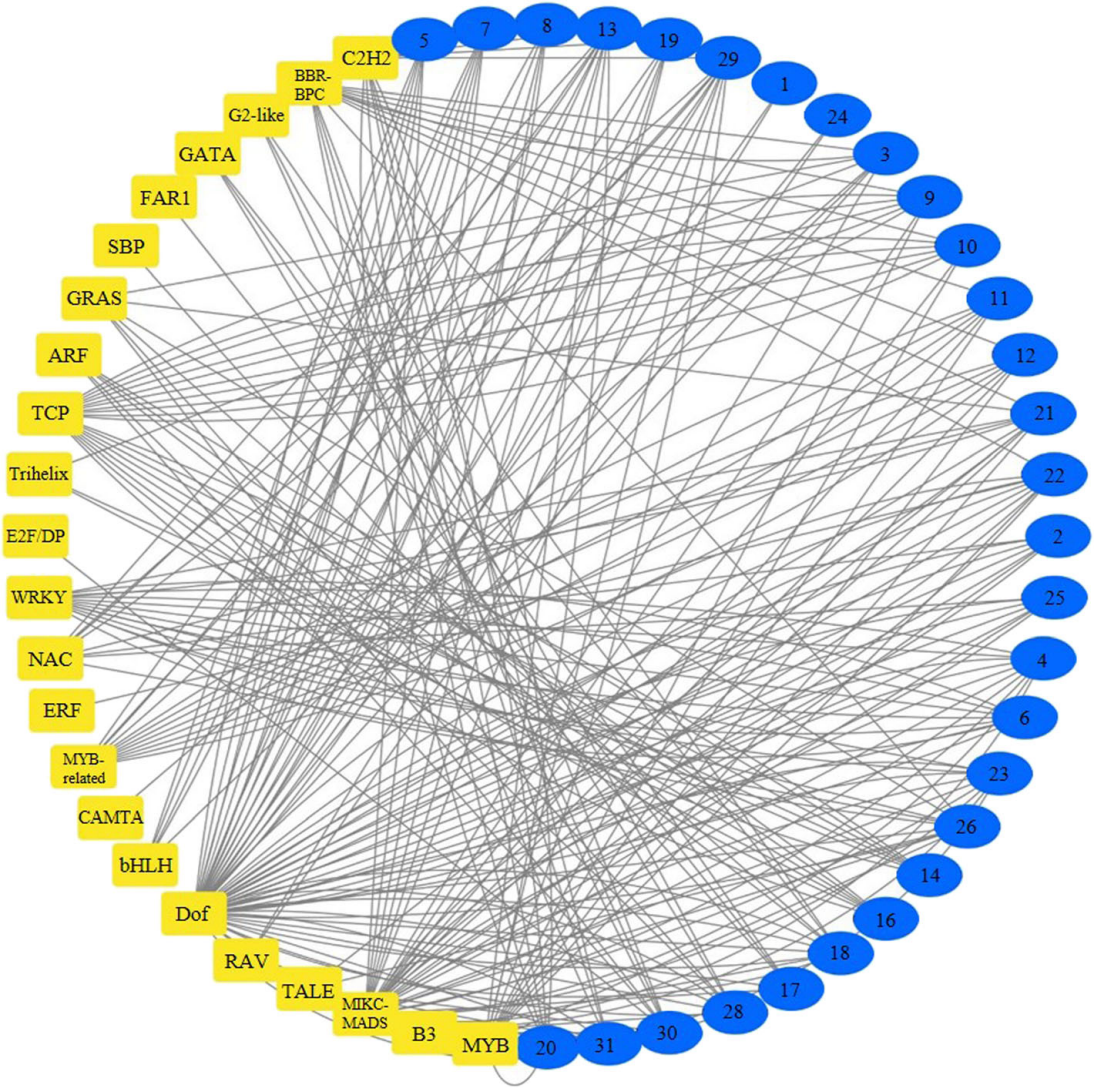
Regulatory network between *NbCrRLK1L* and possible transcription factors. The blue ovals represent *NbCrRLK1L*, the yellow rectangles represent possible transcription factors, and the black lines represent possible regulatory relationships

In order to show the regulatory network of *NbCrRLK1L* in cells more comprehensively, we predicted the possible transcription factors of *NbCrRLK1L*. No corresponding transcription factors were predicted for *NbCrRLK1L15* and *NbCrRLK1L27*, but among the others a total of 23 types of transcription factors were predicted to regulate the expression of this subfamily (Fig. 4 and Additional file 4: Table S4). Among them, Dof, MIKE-MADS, TCP and MYB transcription factors were the most abundant. The different genes were regulated by an average of 7 transcription factors, with *NbCrRLK1L4/13/18/21/22/23/26/28/29* regulated by 10 transcription factors, while *NbCrRLK1L1* and *NbCrRLK1L24* can be regulated by only two (Fig. 4).

### *NbCrRLK1L* tissue-specific expression

**Fig. 5:**
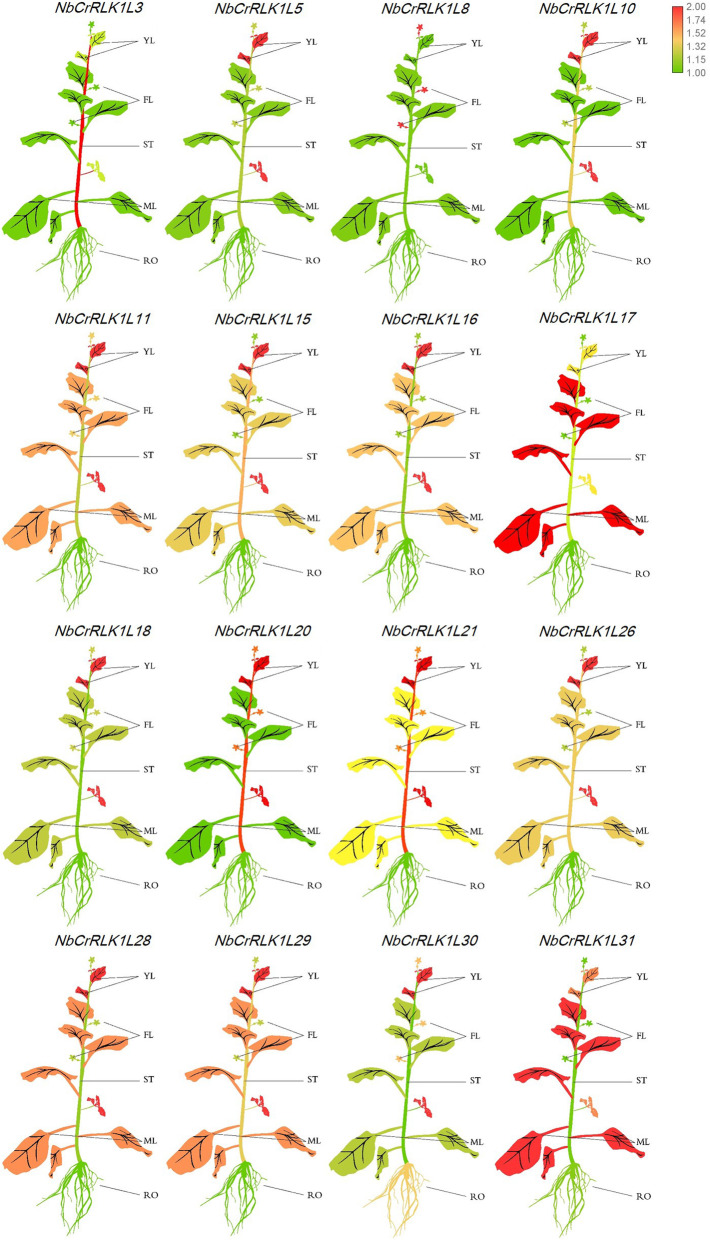
Expression levels of representative *NbCrRLK1Ls* in different tissues. The mean expression value was calculated from three independent biological replicates relative to that in young leaves. YL: young leaf; ML: mature leaf; ST: stem; RO: root; FL: flower. Red represents a high expression level and green represents a low expression level. The raw data of relative expression values and standard errors is provided in Additional file 5: Figure S1

The expression levels of all *NbCrRLK1Ls* were determined in each of the five tissue types (roots, stems, young leaves, mature leaves, flowers), and we here present the results of 16 members that were expressed in all five tissues and which represent each of the phylogenetic groups. Except for *NbCrRLK1L8*, *NbCrRLK1L17* and *NbCrRLK1L31*, the genes were expressed at a higher level in young leaves than in mature ones (Fig. 5 and Additional file 5: Figure S1). *NbCrRLK1L11*, *15*, *16*, *17, 21, 26*, *28*, *29*, *31* are expressed at high levels in leaves, implying that these members may be involved in responding to external stimuli (Fig. 5 and Additional file 5: Figure S1). *NbCrRLK1L8* is expressed at high levels in flowers, *NbCrRLK1L30* is expressed at high levels in roots and *NbCrRLK1L3,* 20, 21 are highly expressed in the stem (Fig. 5 and Additional file 5: Figure S1) showing differences among the genes in their tissue-specific expression patterns.

### Induction of *NbCrRLK1L* expression by pathogens and PAMPs

To explore whether *NbCrRLK1Ls* are involved in the response to pathogens, we inoculated *Pseudomonas syringae* pv *tomato* strain DC3000 (*Pst* DC3000), three viruses (TuMV, PMMoV, PVX) and two PAMPs onto *N. benthamiana* leaves. Two days after *Pst* DC300 infection, 5 days after virus inoculation and 3 h after PAMPs treatment, *N. benthamiana* leaves were collected and RT-qPCR was used to detect the expression patterns of the 16 selected genes.

**Fig. 6:**
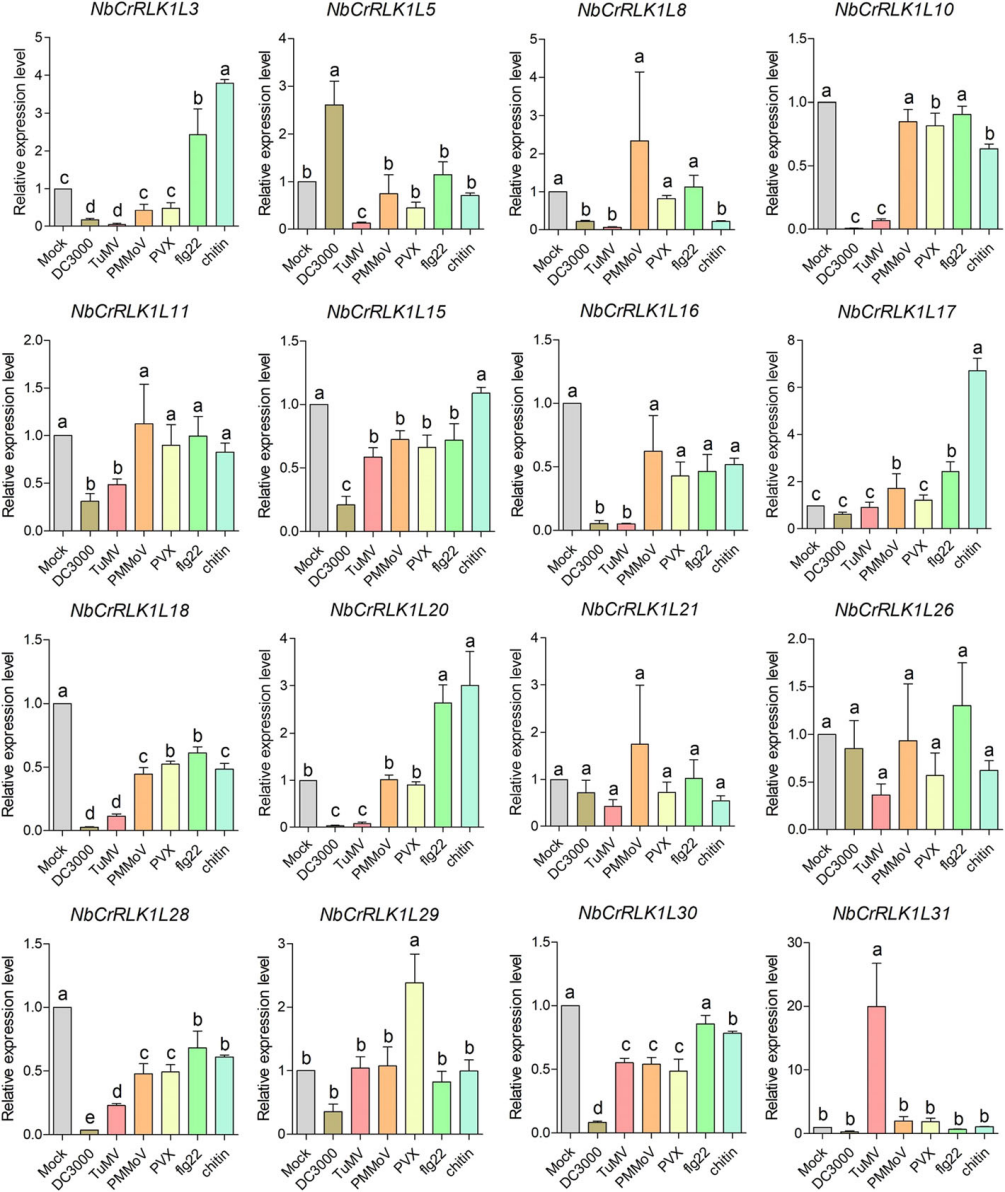
Expression analysis of representative *NbCrRLK1Ls* after treatment of *N. benthamiana* with different pathogens or PAMPs*.* The mean expression values were calculated from three independent biological replicates and were relative to mock-inoculated controls

The bacterial pathogen *Pst* DC300 down-regulated the expression of most genes, especially *NbCrRLK1L3, 8*, *10*, *16*, *18*, 20, *28*, and *30* (Fig. 6). TuMV infection significantly down-regulated these genes, except for *NbCrRLK1L31*, to 0.06–0.55. PMMoV and PVX also down-regulated the expression of some genes, but not to the same extent as *Pst* DC3000 and TuMV. Flg22 and chitin induced the expression of *NbCrRLK1L*3, 17, 20 and slightly down-regulated the expression of some genes, such as *NbCrRLK1L8*, *10*, *18*, and *28* (Fig. 6).

## Discussion

Malectin-like domains are lectin-like motifs found in eukaryotic and prokaryotic proteins. They are particularly abundant in plants and perform very important signal functions in defense and development [7, 37]. The MLD gene has been identified in the genomes of Arabidopsis, rice, cotton, poplar, and strawberry etc. [1, 32–36], but this subfamily has not been identified in *N. benthamiana*. In this study a total of 31 members were identified in *N. benthamiana*. Solis-Miranda et al. identified 16 and 25 CrRLK1L members in *Solanum lycopersicum* and *Solanum tuberosum*, respectively, indicating that there were differences in the number of CrRLK1L genes in different species of the same family [36]. Based on sequence similarity, they could be divided into six groups. Five of the groups have homologous Arabidopsis gene members but the sixth group does not, suggesting that the evolution of this subfamily in different species is independent. Solis-Miranda et al. analyzed the CrRLK1L protein in 57 species and found that members of this subfamily experienced diversification in a wide range of plant groups [36]. We compared the numbers of 11 types of *CrRLK1L* genes in *N. benthamiana*, *Solanum lycopersicum* and *Solanum tuberosum*, and found that *N. benthamiana* had larger numbers of four types of homologous genes (*ANXUR*, *HERKULES1*, *BUPS* and *THESEUS*) than the *Solanum* species, indicating that the first, second, and fifth groups of *NbCrRLK1L* may have expanded. *NbCrRLK1L8/11/18/30* are highly expressed in flowers, which may be related to the function of CrRLK1L subfamily during fertilization. Except for *NbCrRLK1L8*, the other genes were highly expressed in young leaves while *NbCrRLK1L11*/*15*/*16/17/21/26*/*28*/*29*/*31* (and especially *NbCrRLK1L17/31*) were also highly expressed in mature leaves, indicating the functional diversity of subfamily members. These results will provide a basis for further functional classification of NbCrRLK1Ls.

Inoculation with pathogenic bacteria (*Pst* DC3000) and viruses (TuMV, PMMoV, PVX) can inhibit the expression of most of the genes in the NbCrRLK1L subfamily, indicating that NbCrRLK1Ls can respond to pathogen infection. Lindner et al. also found that inoculation with *Pst* DC3000 significantly down-regulated the expression of Arabidopsis CrRLK1Ls [9]. Interestingly, in addition to inhibiting the gene expression of NbCrRLK1Ls, pathogens can also induce expression of certain genes after infection. For example, DC3000 up-regulated the expression of *NbCrRLK1L5* and TuMV up-regulated *NbCrRLK1L31* about 20 fold. The functions of these two genes in the process of infection by these pathogens needs to be explored further.

## Conclusion

This study performs an analysis of the *NbCrRLK1Ls* and provides the basis for a better understand of their varied functions in plant development and in plant-microbe interactions.

## Methods

### Identification of NbCrRLK1L subfamily members

The protein sequences of 17 Arabidopsis CrRLK1Ls were downloaded from TAIR, and the genome of *Nicotiana benthamiana* was downloaded from the Sol Genomics Network (https://solgenomics.net/) [38]. NbCrRLK1Ls were identified by two rounds of BLASTP. First, all Arabidopsis CrRLK1L protein sequences were used to search for possible NbCrRLK1L sequences using TBtools [39]. Then NCBI’s Batch CD-Search function was used to confirm whether the candidate NbCrRLK1Ls had a characteristic malectin-like domain (pfam12819) and kinase domain (cl21453). The candidates that did not meet these conditions were eliminated. The predicted CDS length, PI, and molecular weight of NbCrRLK1Ls were determined by ExPASy [40].

### Phylogenetic analysis

The protein sequences of AtCrRLK1L and NbCrRLK1L were used to construct a phylogenetic tree using the neighbor-joining (NJ) method in Mega X software with 1000 bootstrap replicates. The tree was further annotated using iTOL [41].

### Analysis of conserved motifs, gene structure and functional domains

The conserved motifs of the genes were analyzed by the MEME program [42] with the following parameters: optimal motif width was set to 30–70, the number of repetitions was set to zero or one, the maximum number of motifs was set to identify 10 motifs. Gene structure and functional domains were analyzed and visualized using NCBI Batch CD-Search and TBtools.

### Prediction of promoter cis-acting elements and transcription factors

Promoter cis-acting elements were predicted by PlantCARE [43] and visualized by TBtools. Transcription factors were predicted by PlantRegMap [44], and *N. sylvestris* was set as the target analysis species. Cytoscape 3.7.1 was used to visualize the target relationship between transcription factors and NbCrRLK1Ls [45].

### Pathogen inoculation and PAMPs treatment

Plant growth conditions were as described previously [46]. The concentration of TuMV, PMMoV agrobacterium solution was adjusted to OD_600_ = 0.1, and the concentration of PVX agrobacterium solution was adjusted to OD_600_ = 1 × 10^− 4^. The *N. benthamiana* leaves were injected with a needleless syringe, and the leaves injected with the transient transfection solution were used as a blank control. *Pst* DC3000 was cultured on King’s B medium at 28 °C for 2 days. The concentration of *Pst* DC3000 suspension was adjusted to OD_600_ = 1 × 10^− 5^ to infiltrate the leaves, and the leaves injected with 10 mM MgCl_2_ were used as a blank control [46]. 1 μm flg22 and 200 μg/mL chitin dissolved in deionized water, containing 0.01% Silwet L-77, were evenly sprayed on the fully extended *N. benthamiana* leaves, and the leaves only sprayed with deionized water were used as controls. The flg22 peptide was synthesized by Sangon Biotech (Shanghai).

### Gene expression analysis

Total RNA was extracted by the TRIZOL method, and 1 μg total RNA was used for reverse transcription using the Toyobo cDNA First Strand Synthesis Kit. RT-qPCR was then performed on a Roche LightCycler®480 Real-Time PCR instrument with Toyobo Premix Kit. Three independent biological replicates with three technical replicates were performed. All primers are listed in the Additional file 6: Table S5.

## Supplementary Information

**Additional file 1: Table S1.** List of NbCrRLK1L protein sequences.

**Additional file 2: Table S2.** The MEME motif sequences and lengths of NbCrRLK1Ls.

**Additional file 3: Table S3.** Cis-acting elements in *NbCrRLK1Ls*.

**Additional file 4: Table S4.** Potential transcription factors of *NbCrRLK1Ls*.

**Additional file 5: Figure S1.** Expression levels of representative *NbCrRLK1Ls* in different tissues (raw data).

**Additional file 6: Table S5.** Primers used in this study.

## Data Availability

All data generated or analyzed during this study are included in this published article and its additional files. The datasets used and analyzed during the current study are available from the corresponding author on reasonable request. The analysis websites used in this study are as follows: Batch CD-Search (https://www.ncbi.nlm.nih.gov/Structure/bwrpsb/bwrpsb.cgi), ExPASy (https://www.expasy.org/), iTOL (https://itol.embl.de/index.shtml), MEME (http://meme-suite.org/tools/meme), PlantCARE (http://bioinformatics.psb.ugent.be/webtools/plantcare/html/), PlantRegMap (http://plantregmap.gao-lab.org/network.php). The public’s access to these databases is open and there is no sequencing data in this study.
