# Supplementary figures and images for: Genome-wide identification and analysis of Catharanthus roseus RLK1-like kinases in Nicotiana benthamiana
Source: BMC Plant Biol. 2021 Sep 18;21:425. doi: 10.1186/s12870-021-03208-x (PMC8449480; doi:10.1186/s12870-021-03208-x)

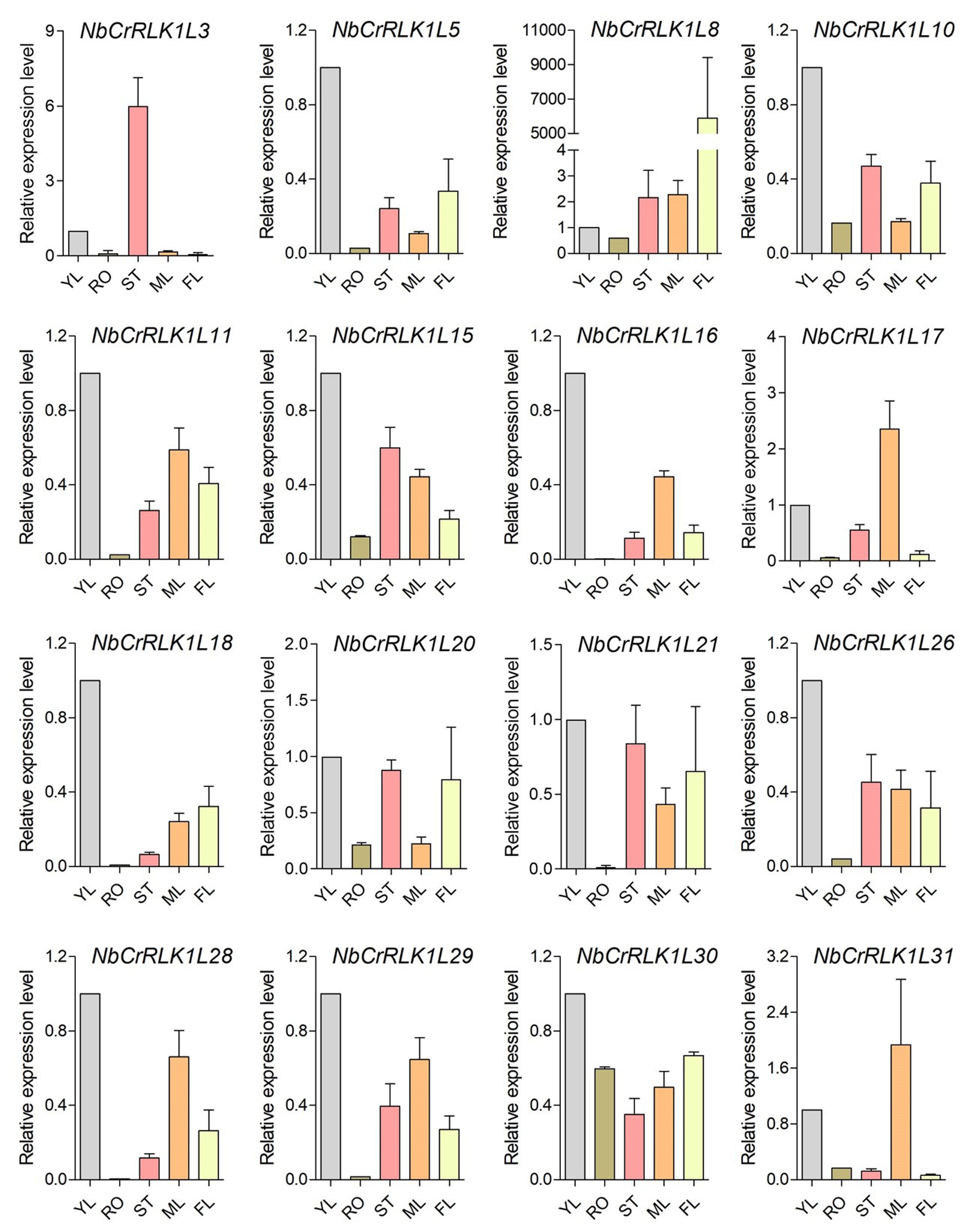

Supplement: Supplementary file 5 — Additional file 5: Figure S1. Expression levels of representative NbCrRLK1Ls in different tissues (raw data). [file 12870_2021_3208_MOESM5_ESM.jpg]
